# *PATRONUS1* is expressed in meiotic prophase I to regulate centromeric cohesion in Arabidopsis and shows synthetic lethality with *OSD1*

**DOI:** 10.1186/s12870-015-0558-6

**Published:** 2015-08-14

**Authors:** Dipesh Kumar Singh, Charles Spillane, Imran Siddiqi

**Affiliations:** Centre for Cellular and Molecular Biology (CSIR), Uppal Road, Hyderabad, 500007 India; Genetics and Biotechnology Lab, Plant and AgriBiosciences Research Centre (PABC), Botany and Plant Sciences, School of Natural Sciences, National University of Ireland Galway, University Road, Galway, Ireland

**Keywords:** Centromere, Kinetochore attachment, Spindle, Anaphase promoting complex (APC/C)

## Abstract

**Background:**

Retention of sister centromere cohesion during meiosis I and its dissolution at meiosis II is necessary for balanced chromosome segregation and reduction of chromosome number. *PATRONUS1 (PANS1)* has recently been proposed to regulate centromere cohesion in *Arabidopsis* after meiosis I, during interkinesis. *pans1* mutants lose centromere cohesion prematurely during interkinesis and segregate randomly at meiosis II. PANS1 protein interacts with components of the Anaphase Promoting Complex/Cyclosome (APC/C).

**Results:**

We show here that PANS1 protein is found mainly in prophase I of meiosis, with its level declining late in prophase I during diplotene. PANS1 also shows expression in dividing tissues. We demonstrate that, in addition to the previously reported premature loss of centromere cohesion during interkinesis, *pans1* mutants show partially penetrant defects in centromere cohesion during meiosis I. We also determine that *pans1* shows synthetic lethality at the level of the sporophyte, with *Omission of Second Division 1* (*osd1*), which encodes a known inhibitor of the APC/C that is required for cell cycle progression during mitosis, as well as meiosis I and II.

**Conclusions:**

Our results show that PANS1 is expressed mainly in meiosis I where it has an important function and together with previous studies indicate that PANS1 and OSD1 are part of a network linking centromere cohesion and cell cycle progression through control of APC/C activity.

**Electronic supplementary material:**

The online version of this article (doi:10.1186/s12870-015-0558-6) contains supplementary material, which is available to authorized users.

## Background

Controlled release of sister chromatid cohesion is essential for balanced segregation of chromosomes in mitosis and meiosis. Cohesion is brought about by means of the cohesin complex, a ring-shaped structure that is thought to encircle sister chromatids. Loading of cohesin takes place during telophase or early in G1 and is followed by establishment of cohesion during S phase [1] The cohesin complex is comprised of four subunits that are conserved across species. The kleisin subunit of cohesin Sister chromatid cohesion 1/ Radiation sensitive 21 (Scc1/Rad21) in mitosis and the meiotic variant Recombination defective 8 (Rec8) is responsible for closing the cohesin ring. Cleavage of the kleisin subunit at the metaphase to anaphase transition by the separase protease allows separation of sister chromatids at mitosis [2–4]. In meiosis, unlike in mitosis, sister centromere cohesion is retained through meiosis I until metaphase of meiosis II. SHUGOSHIN (SGO) and protein phosphatase 2A (PP2A) play an important role in controlling centromere behavior during meiosis [5–8]. During meiosis I, SGO is responsible for recruitment of protein phosphatase PP2A to centromeres which keeps REC8 unphosphorylated and resistant to cleavage by separase. During meiosis II, REC8 is no longer protected and at the metaphase to anaphase transition, cleavage of REC8 by separase leads to separation of sister chromatids [9–11].

Unlike in yeast, during mitosis in higher eukaryotes most of the cohesin located along chromosome arms is released during prophase by a mechanism involving its phosphorylation by Polo kinase and additional proteins including WAPL helicase [12–16]. However, centromeric cohesin is protected from release during prophase of mitosis by the action of SGO and PP2A. SGO proteins have also been found to play a role in kinetochore orientation [17] and are essential for viability in mice [18]. SGO proteins therefore regulate both kinetochore structure/orientation in addition to their role in protecting centromere cohesion during prophase of mitosis and in meiosis I divisions.

Recently the *PATRONUS* (*PANS1*) gene has been shown to be required for maintenance of sister chromatid cohesion during interkinesis of meiosis in *Arabidopsis thaliana* leading to the proposal that sister centromere cohesion is protected at two stages during meiosis: during anaphase I, by the action of SGOs and following that during interkinesis by PANS1 [19, 20]. We further investigated the function of *PANS1* and demonstrate here that PANS1 protein is present maximally in meiosis I and distributed broadly across the nucleus. We determine that, in addition to the major phenotype of *pans1* comprising loss of sister chromatid cohesion in meiosis II, *pans1* mutant meiocytes also show subtle differences in centromere organization in meiosis I. We further find that *pans1* shows synthetic lethality with *osd1*, which encodes an inhibitor of the APC/C ubiquitin ligase that has been shown to be required for progression through meiosis and entry into meiosis II [21]. Our results indicate that PANS1 acts through control of the APC/C and that PANS1 is part of a network that includes APC/C and OSD1, that modulates meiotic progression and sister chromatid cohesion, possibly through control of ubiquitination. The timing of expression as well as the meiosis I phenotypes we observe suggest that PANS1 acts in meiosis I.

## Results

### Centromere phenotypes in meiosis I and loss of centromere cohesion during meiosis II in *pans1* mutants

**Fig. 1:**
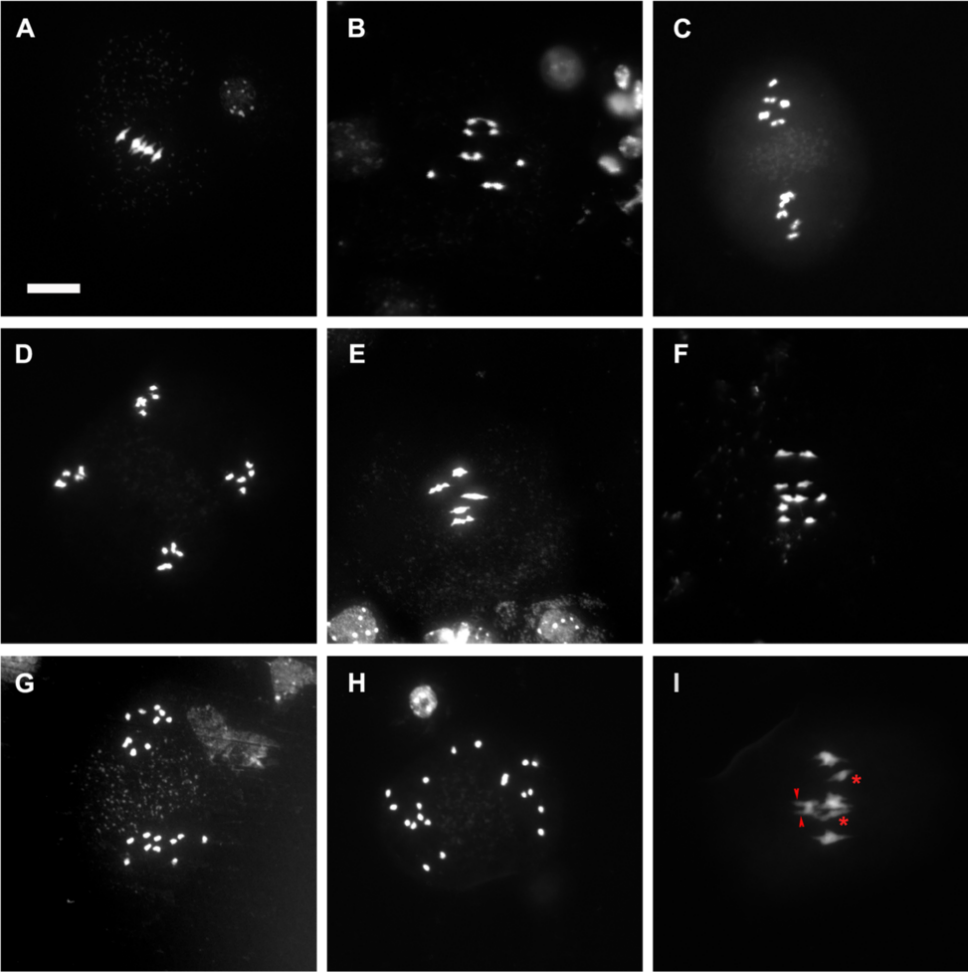
Early loss of centromere cohesion in *pans1* during meiosis II. Acid spreads of male meiotic chromosomes stained with DAPI. **a-d** wild type, **e-i** *pans1*. **a,e** Normal metaphase I. **b, f** Normal anaphase I. **c** Normal metaphase II **d** Normal anaphase II. **g** Early loss of cohesion in *pans1* meiosis II prior to metaphase II. **h** Random movement of chromosomes in meiosis II in *pans1*. **i** *pans1* defective metaphase I showing bipolar attachment (arrowheads) and univalents (*) in a subset of chromosomes

*pans1* mutants of Arabidopsis have been recently reported to cause reduced fertility that arises from a defect in maintenance of centromeric cohesion during interkinesis of male meiosis [19, 20]. In *pans1*, chromosomes have been reported to undergo a normal reductional segregation at meiosis I, however during meiosis II, chromosomes lose centromeric cohesion prematurely prior to metaphase and as a consequence segregation occurs randomly resulting in the formation of unbalanced meiotic products and reduction in fertility. We confirmed the interkinesis phenotype of *pans1* comprising loss of centromeric cohesion prematurely in meiosis II, prior to metaphase leading to unbalanced segregation (Fig. 1) and formation of defective microspores (Additional file 1: Figure S1): at metaphase I the majority of *pans1* meiocytes showed 5 bivalents as in wild type; in contrast at metaphase II all (86/86) *pans1* meiocytes showed 6–10 chromosomes indicating separation of sister chromatids, whereas for wild type, no meiocytes showed separation of sister chromatids at metaphase II (Table 1). Hence the major phenotype of *pans1* is in meiosis II.

**Table 1:** Meiosis I and Meiosis II phenotypes in the pans1 mutant

Stage	Phenotype	Wild Type	*pans1* mutant
Metaphase I	univalents	0/30	5/32 = 15 %
Metaphase I	Splitting of sister centromere FISH signal	0/28	8/38 = 21 %
Metaphase II	Separated sister chromatids	0/8	86/86 = 100 %

While the majority of *pans1* meiocytes showed normal pairing, alignment, and segregation in meiosis I, we also observed a partially penetrant meiosis I phenotype in *pans1* comprising the presence of two or more univalents at metaphase I (Fig. 1I; Table 1). Univalents were not observed at metaphase I in the case of wild type. Hence the novel meiosis I phenotype of *pans1* described here is significant (*X*^2^ = 6.0; p < 0.05). Previous studies have described *pans1* as having a meiotic phenotype confined to interkinesis on the basis of which PANS1 has been proposed to specifically control centromeric cohesion during interkinesis [19, 20].

**Fig. 2:**
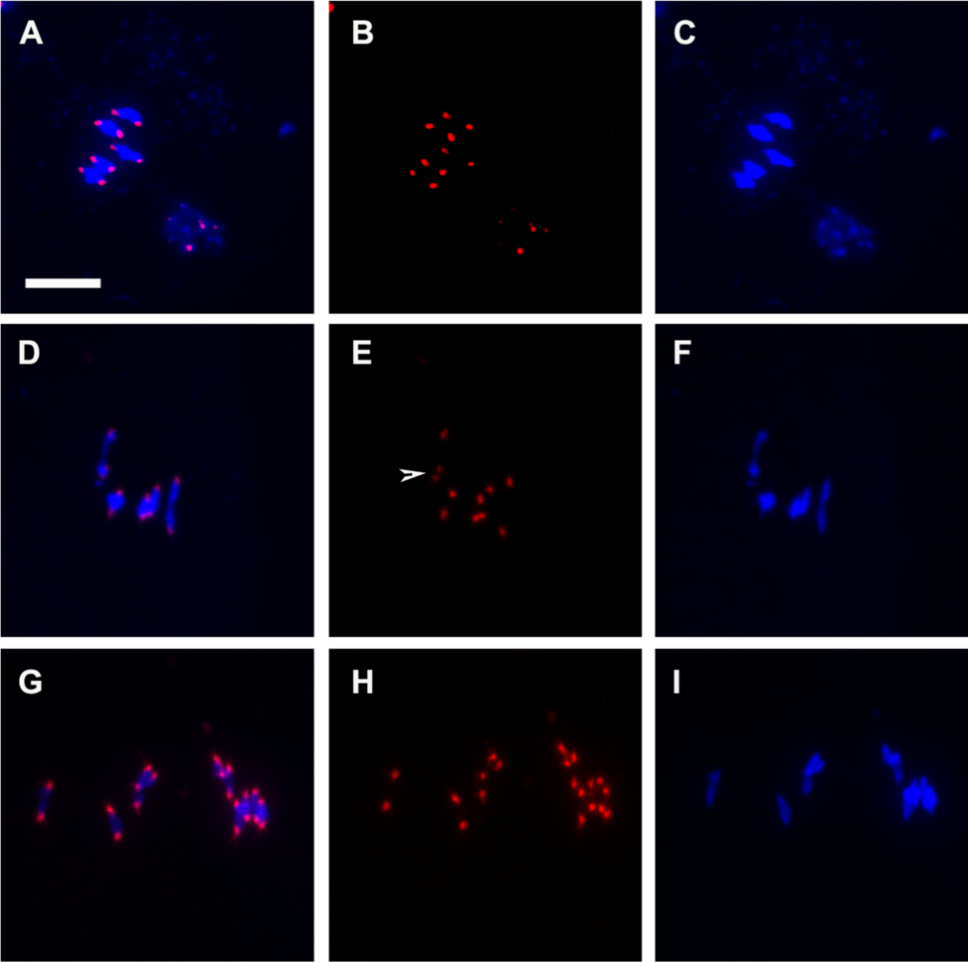
Loss of centromeric cohesion in *pans1* male meiocytes during Meiosis I. FISH on male meiotic chromosome spreads using a centromeric repeat probe showing DAPI (*blue*) and probe (*red*). Left column: merged images of DAPI and the probe; middle column: probe alone; right column: DAPI. **a-c** wild type metaphase I. **d-i** *pans1* metaphase I. **d-f** mild phenotype showing split centromere signal on one chromosome indicated by arrow head. (**g-i**) strong phenotype showing univalents of one chromosome and split centromere signals on four chromosomes. Scale bar 10 μm

Loss of centromeric cohesion in *pans1* during meiosis II could be due to defects in regulation of cohesion specifically during meiosis II, or alternatively could also be connected to defects in centromere organization during meiosis I. The small number of meiocytes that we observed exhibiting a phenotype in meiosis I prompted us to further examine centromere organization. To probe centromere structure during meiosis I, we carried out fluorescence in situ hybridization on meiotic chromosome spreads using a pAL1 centromere repeat probe that hybridizes to pericentromeric repeats [22]. Centromeres in wild type gave regular and compact signals at metaphase I and showed five bivalents, with two signals per bivalent, each signal representing a pair of sister centromeres. In contrast, differences were observed for *pans1* wherein about 21 % of the metaphase I stages showed four centromere signals in a bivalent (Fig. 2; Table 1), indicating that sister centromeres were not closely connected. We did not observe splitting of sister centromere signals in midprophase I from zygotene to pachytene in *pans1* (0/102 meiocytes; Additional file 1: Figure S2). Our interpretation is that although the sister centromeres are still connected at metaphase I, the connection is not as tight as for wild type and separation of the sister centromere signals occurs following attachment of sister centromeres to the meiosis I spindle. The presence of two or more univalent chromosomes was also seen in 5/8 meiocytes showing split centromere signals at metaphase I and the univalent chromosomes showed bipolar attachment to the meiosis I spindle (Fig. 2G-I). A mixture of reductional and equational segregation therefore appears to be taking place in these meiocytes. These results indicate that in addition to the major phenotype which reflects a requirement for PANS1 in retention of centromere cohesion after meiosis I and up to metaphase of meiosis II (in agreement with earlier reports [19, 20]) there is a requirement for PANS1 in centromere cohesion during meiosis I, as reflected in a partially penetrant *pans1* phenotype with regard to centromere cohesion in meiosis I.

### PANS1 is expressed in dividing tissues, during meiotic prophase, and displays broad nuclear localization

**Fig. 3:**
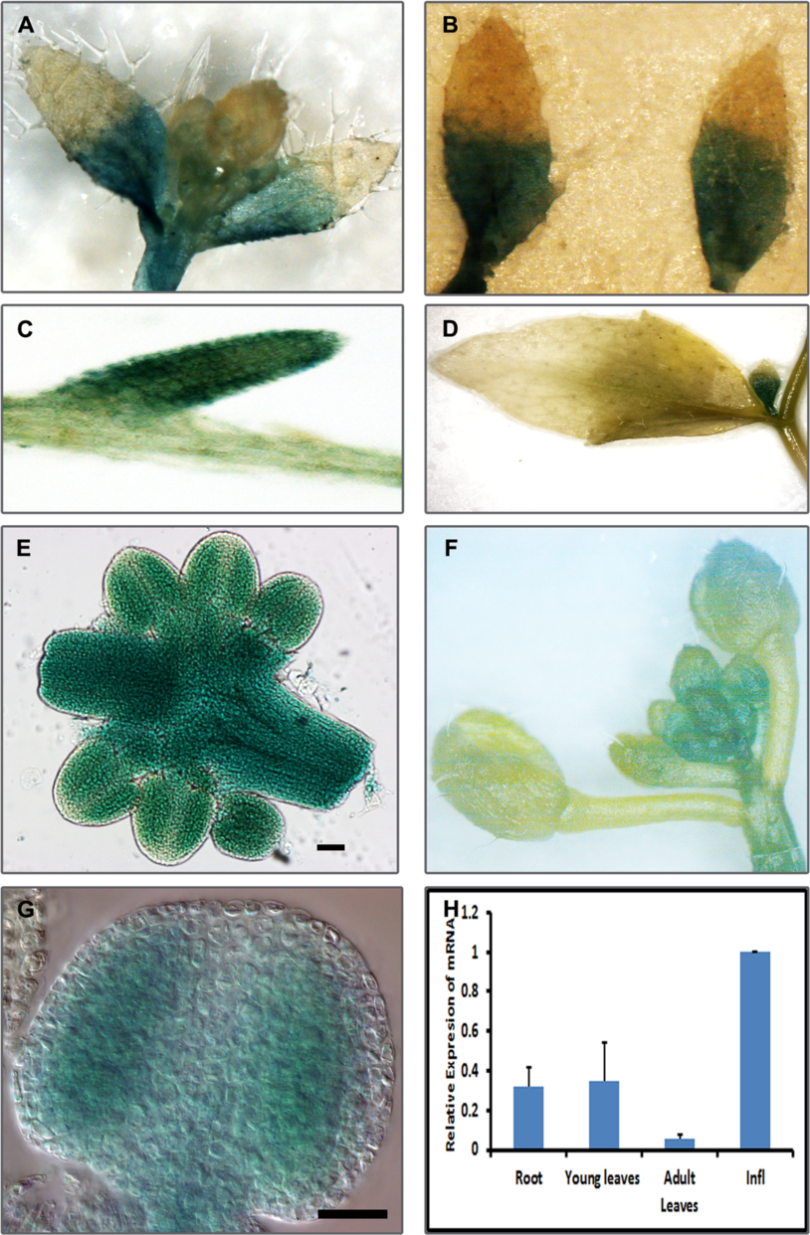
PANS1 is expressed in actively dividing tissue. *P*_*pans1.*_ *PANS1-GFP-GUS* fusion plants shows GUS expression in dividing tissues. **a** Shoot meristematic region and cauline leaves. **b** Young cauline leaves showing GUS expression in basal portion. **c** Root **d** GUS expression in axillary buds but not in adult cauline leaves. **e** High levels of expression in developing anthers and pistil. **f** Inflorescence **g** Meiotic stage anther. **h** Quantitative reverse transcription PCR (q-RT-PCR) of PANS1. Columns indicate the mean of levels of expression, error bars indicate standard deviation. Scale bar represents 100 μm in (**e**) and 20 μm in (**g**)

A genomic fragment comprising the PANS1 promoter and coding region was fused to a composite Green Fluorescent Protein-Beta Glucuronidase (GFP-GUS) reporter to generate a fusion protein and the transgene cassette transformed into plants. Analysis of GUS reporter gene expression indicated that PANS1 shows increased expression in growing parts of the plant, being expressed in inflorescence, young buds, and roots (Fig. 3). Expression declined in older buds and was observed in the basal but not distal portion of young leaves coincident with the pattern of cessation of cell division in leaves which proceeds from tip to base. Quantitative analysis of gene expression indicated that PANS1 is strongly expressed in the inflorescence and at a lower level in leaves and in roots.

**Fig. 4:**
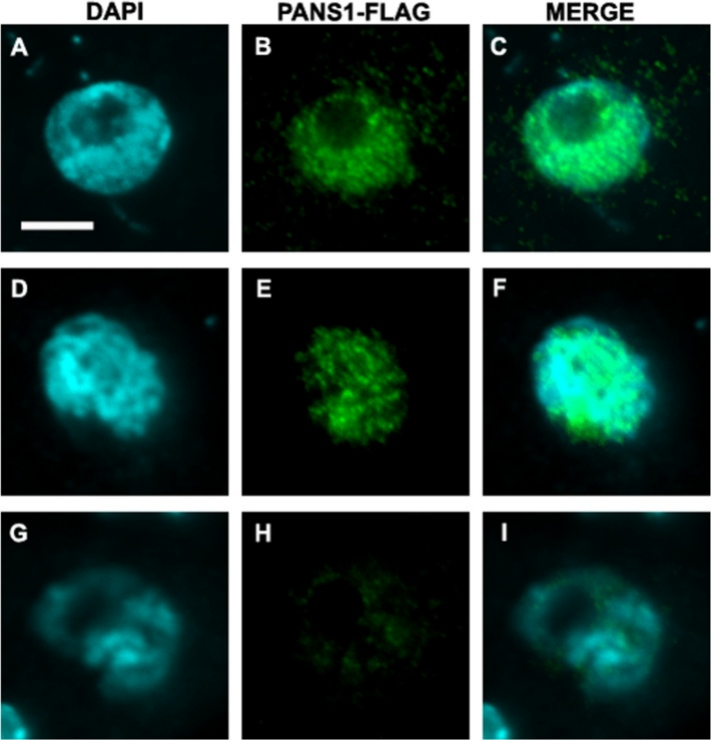
PANS1-FLAG is localized to the nucleus during prophase I of Meiosis. Immunostaining of anther squashes of PANS1-FLAG transgenic line, showing chromosomes stained with DAPI (*cyan*), FLAG (*green*) and merged images for cyan and green channels (*right column*)**.** **a-c** Early prophase I, **d-f** Mid prophase I, **g-i** Late prophase I

To obtain more detailed information on subcellular localization of PANS1 in meiocytes, we generated a FLAG-tagged PANS1 line in a *pans1* mutant background in which the *pans1* mutant was complemented by the FLAG-tagged allele. We then examined protein localization in meiosis using an anti-FLAG antibody. A strong PANS1-FLAG signal was observed up to mid-prophase and declined during late prophase (Fig. 4; Additional file 1: Figure S3). The pattern of localization extended broadly across the nucleus but appeared to be excluded from the nucleolus. The timing of maximal PANS1 expression during meiosis therefore appears to precede the onset of the mutant phenotype (comprising defects in centromere organization starting late in meiosis I and extending to interkinesis during the second meiotic division).

### *pans1* shows synthetic lethality with *osd1*

**Fig. 5:**
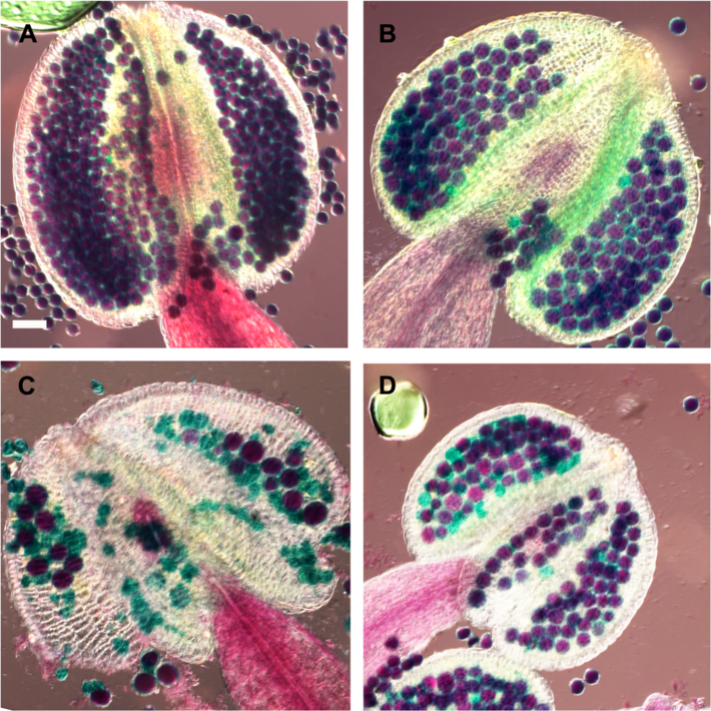
*tam1-2* suppresses *pans1* sterility. Pollen viability by Alexander staining. **a** wild type. **b** *tam1-2*. **c** *pans1-1* **d** *tam1-2/pans1-1.* Scale bar represent 50 μm

To test whether the sterility caused by *pans1* is due primarily to defective chromosome segregation during meiosis II, we crossed *pans1* with (hemizygous) *tardy asynchronous meiosis 1* (*tam1*) and *osd1* mutants in which the majority of meiocytes do not undergo the second meiotic division, leading to the formation of unreduced gametes [23–25]. In the case of the *pans1 x tam1* crosses we obtained 9 double mutant plants out of 180 total examined in the F2 (Table 2). All of the *pans1 tam1* double mutant plants showed an increase in viable pollen and seed set compared to the *pans1* mutant, indicating genetic suppression of the male sterile phenotype of *pans1* by a *tam1* loss of function allele (Fig. 5; Additional file 1: Figure S4; Table S1). The minority class of inviable pollen (green) observed in *pans1 tam1* can be explained by the minority class of meiocytes that undergo meiosis II in *tam1* [23, 25].

**Table 2:** Genetics of pans1 interaction with tam1 and osd1

Parent/Cross	No. of progeny and genotype/phenotype				Total no.
pans1/PANS1 tam1/TAM1	*PANS1/- TAM1/-*	*PANS1/- tam1*	*pans1 TAM1/-*	*pans1 tam1*	
	*111* fertile	*32* fertile	*28* sterile	*9* fertile	180
pans1/TAM1 osd1/OSD1	*PANS1/- OSD1/-*	*PANS1/- osd1*	*pans1 OSD1/-*	*pans1 osd1*	
	*168*	*35*	*36*	*0*	239
pans1/PANS1 osd1/OSD1 X WT	*WT*	*pans1/PANS1*	*osd1/OSD1*	*pans1/PANS1 osd1/OSD1*	
	*15*	*15*	*15*	*9*	54
WT X pans1/PANS1 osd1/OSD1	*WT*	*pans1/PANS1*	*osd1/OSD1*	*pans1/PANS1 osd1/OSD1*	
	*14*	*14*	*15*	*9*	52

When *pans1* was crossed to *osd1* we failed to obtain any *pans1 osd1* double mutants (Table 2; P < < 0.001). The failure to obtain the *pans1 osd1* double homozygous mutant is indicative of a synthetic lethal interaction between *pans1* and *osd1* and provides robust evidence that the two genes have related functions. To determine if the synthetic lethality is gametophytic or sporophytic we carried out reciprocal crosses between *pans1/+ osd1/+* and wild type. In both crosses we obtained *pans1/+ osd1/+* F1 plants indicating that both male and female *pans1 osd1* gametes are viable and functional and that therefore the synthetic lethality is a sporophytic phenotype (Table 2). However in the *pans1/+ osd1/+* X WT cross, we observed a small but significant reduction in seed set (6.9 ± 1.3 missing seeds per silique) when compared to the WT X WT cross (0.7 ± 0.78 missing seeds per silique which points to reduced fitness of *pans1 osd1* female gametes.

Both OSD1 and PANS1 have been shown to interact with components of the APC/C that regulates the metaphase to anaphase transition [19, 21, 26] and cell cycle progression through control of proteasome mediated degradation. In addition to a role in meiosis, OSD1 and PANS1 also function in mitosis to control chromosome ploidy [27, 28]. Our results provide evidence that PANS1 and OSD1 are part of a network that functions with APC/C to link centromere structure and sister chromatid cohesion with cell cycle progression.

## Discussion

PANS1 has been reported to control sister centromere cohesion during interkinesis between meiosis I and meiosis II [19, 20] in Arabidopsis. We show here that *pans1* also has a meiosis I phenotype with regard to split sister centromere signals at metaphase I and sporadic formation of univalents. These observations together with the maximal presence of PANS1 protein in prophase I of meiosis that we describe here indicate that PANS1 plays an important role in control of sister chromatid cohesion during meiosis I as well, and that the function of PANS1 during meiosis is not limited to meiosis II.

Approximately 21 % of metaphase I stages in *pans1* showed greater than 10 centromere signals with one or more bivalents showing 4 signals. Split sister centromere signals were never observed in wild type indicating that the difference is significant. Formation of univalents of one or more chromosomes was also observed in some cases (15 % of metaphase I stages) in *pans1*, and chromosomes showed a mixture of monopolar and bipolar attachment. In *Arabidopsis thaliana*, REC8 and SISTER CHROMATID COHESION 3 (SCC3) cohesins are required for both centromere cohesion as well as monopolar attachment of sister centromeres in meiosis I and *Atrec8* and *Atscc3* mutants show loss of centromere cohesion and bipolar attachment of sister kinetochores [29]. The occurrence of split sister centromere signals at metaphase I in a significant proportion of *pans1* meiocytes is indicative of a difference in the closeness of connection between sister centromeres relative to wild type. Chromosomes appear to show mixed segregation in this class of *pans1* meiocytes. The reduced strength of centromeric cohesion in *pans1* could possibly arise from a lowered amount of cohesin at the centromeric region. This possibility is supported by the formation of univalents that occurs sporadically in *pans1* which is also observed in cohesin mutants [29].

PANS1 is expressed primarily in dividing cells and the protein localizes to the nucleus. Nuclear localization of PANS1 in cultured cells has also been described previously [30]. PANS1 also shows expression during meiosis and the protein is present at early prophase I where it can be detected broadly across the nucleus but is excluded from the nucleolus. The protein signal declines late in prophase I at the diplotene stage. PANS1 has two degradation motifs: a DEN-box and a D-box which have been shown to be important for its normal function [19]. The presence of the protein in prophase of meiosis I correlates with the partially penetrant phenotypic differences that we observed with regard to altered centromeric cohesion in a class of *pans1* meiocytes at metaphase I.

SGO and PP2A are located at (and act directly on) centromeres to protect Rec8 from cleavage by Separase during meiosis I thereby preserving centromere cohesion in meiosis I [6]. PANS1 on the other hand shows a broad distribution throughout the nucleus with a maximal signal found in prophase I and decreasing in late prophase. Loading of APC/C at the centromeres by the spindle assembly checkpoint has been shown for mitosis in human cells and this may be the form that is relevant for control of cohesion at the centromere in plants as well [31]. The occurrence of PANS1 throughout the nucleus in meiosis prophase I could reflect a role in other aspects of APC/C function (see below) and PANS1, also called COPPER MODIFIED RESISTANCE 1 (CMR1) has been shown to be involved in response to stress [30]. We did not detect PANS1 protein in meiosis II by immunostaining, however the possibility that a small amount of protein is still present in meiosis II and regulates centromere cohesion during interkinesis is not ruled out. Hence PANS1 may function independently in both meiosis I (based on immunostaining results along with the meiosis I phenotypes described above) as well as in meiosis II where it controls centromeric cohesion during interkinesis. Alternatively PANS1 may control levels of a factor in meiosis I that acts later during interkinesis to control centromeric cohesion. In fact as noted above, the timing of maximal PANS1 expression precedes the onset of the meiosis I and meiosis II phenotypes.

PANS1 has been shown to interact with components of the APC/C CELL DIVISION CYCLE 27b (AtCDC27b)/HOBBIT and CELL DIVISION CYCLE 20.1 (CDC20.1) [1] as well, suggesting that it controls centromere organization and cohesion during meiosis through regulation of the APC/C. Centromeric cohesion is resistant to dissolution by separase during meiosis I but not during meiosis II. The difference is thought to be an intrinsic property of the chromosomes since placement of meiosis I chromosomes onto a meiosis II spindle and vice versa does not change the behavior of the chromosomes [32]. One possible route of action of PANS1 would be by specifically controlling factors such as Separase, Securin, Shugoshin, PP2A, and WAPL, that are responsible for protection or removal of cohesin [8, 16, 19, 20, 33]. A second possibility is that PANS1 may control kinetochore proteins such as MINCHROMOSOME SEGREGATION 12 (MIS12) whose depletion in maize is known to lead to bipolar attachment [34, 35]. Alternatively, the loss of cohesion in *pans1* may be a consequence of broader changes in regulation of the cell cycle arising from altered APC/C function affecting centromere properties. Recent evidence from yeast has shown that deregulation of the cell cycle during meiotic prophase I leads to disruption in sister kinetochore co-orientation and in protection of centromere cohesion [36]. Changes in regulation of the meiotic cell cycle could likewise be responsible for the centromere phenotypes in the case of *pans1*, covering both meiotic divisions.

We found that *pans1* shows synthetic lethality with *osd1*. OSD1 is required for entry into both meiosis I and meiosis II divisions [25] as well as for control of ploidy in mitosis [26] and has been proposed to be an inhibitor of the APC/C, regulating both mitotic and meiotic progression as well as showing protein-protein interaction with APC/C activators including CDC20.1 and CDC20.5, [21, 27]. PANS1 protein has also been shown to interact with the APC/C components CDC20.1, and CDC27b although the same study did not identify OSD1 in a tandem affinity purification experiment using PANS1 as bait [19]. Hence the interaction of PANS1 with OSD1 may be indirect and not at the protein-protein level. One explanation for the observed synthetic lethality between PANS1 and OSD1 is that PANS1, like OSD1, is an inhibitor of the APC/C and that loss of both OSD1 and PANS1 together leads to a highly deregulated APC/C that results in lethality.

## Conclusions

We conclude that PANS1 acts in meiosis I in addition to having a role in meiosis II and that PANS1 and OSD1 are part of a network that links centromere cohesion and cell cycle progression through control of the APC/C via interactions with APC/C regulators and core APC/C components. Our results highlight the importance of coordinated APC/C control for orchestration of chromosome segregation and cell cycle progression as well as cell viability.

## Methods

### Plant materials and growth conditions

The *Arabidopsis thaliana* strains used were of the Columbia ecotype (Col-0). The T-DNA insertion lines salk_035661 (*pans1-1*), salk_070337 (*pans1-2)* and sail_505 (*tam1-2*) were obtained from Nottingham Arabidopsis Stock Centre (NASC) and Arabidopsis Biological Resource Center (ABRC). The *osd1-3* mutant was kindly provided by Raphael Mercier, INRA, France. Plants were grown as described earlier [37]. Transgenic plants for 2XFLAG tag, PANS1-GFP-GUS gene fusion and complementation were selected on MS media containing glufosinate ammonium (Sigma) 10 μg/ml with 2 % sucrose.

### Genetic and functional analysis

The presence of a T-DNA insertion in *PANS1* was determined by PCR using a left-border outwardly directed primer (SALK_LB1.3) in combination with a gene-specific primer flanking the site of insertion. SALK_035661-RP and SALK_LB1.3 primers were used to test for insertion of both *pans1-1* and *pans1-2* and SALK_035661-RP and SALK_035661-LP for wild type. To test for allelism and for double mutant analysis, plants homozygous for *pans1-1 were* crossed with heterozygous *pans1*-*2*, *osd1-3*, and *tam1-2* (sail_505) single mutants and F1 and F2 plants were genotyped according to [21].

### Real time PCR

Total RNA was isolated using Trizol (Invitrogen) as per the manufacturer’s protocol. cDNA was synthesized from 2 μg of total RNA using the Superscript III first strand cDNA synthesis kit (Invitrogen) with Oligo (dT) primers. Real Time PCR reactions were done in a 10 μl volume comprising of primer, cDNA template and 1× SYBR Green PCR master mix (Applied Biosystems). GAPC was used as the internal normalization control. PCR was performed on the ABI Prism 7900 HT Fast Real-time PCR Sequence Detection System (Applied Biosystems) in a 384 well reaction plate according to the manufacturer’s recommendations. Primers were F_PANS1qRT and R_PANS1qRT for *PANS1* and F_GAPCqRT and R_GAPqRT for *GAPC*. Cycling parameters consisted of 2 min incubation at 50 °C, 10 min at 95 °C and 40 cycles of 95 °C for 15 s, 57 °C for 30 s and 68 °C for 30 s. Each PCR reaction was performed in three technical replicates across four biological replicates. Specificity of the amplifications was verified at the end of each PCR run using ABI prism dissociation curve analysis. Quantification of mRNA was determined from threshold cycle (Ct values) obtained in the log-linear range of real time PCR amplification plots [38]. The Mann–Whitney *U* test performed on mean ΔCt values indicated that the leaf and root samples were significantly different from the inflorescence samples (p < 0.05).

### Preparation of constructs

To study PANS1 localization, a PANS-2xFLAG construct was prepared by amplifying genomic DNA from Col-0 comprising 1006 bp upstream of the ATG upto the last amino acid coding sequence using PANS1FL-F and PANS1cflag-R primer containing FLAG sequence. The PCR fragment was cloned into pENTR/D-TOPO vector (Invitrogen) and then mobilized into C-ter FLAG destination vector pEARLY 302 [39] by LR reaction.

To prepare a PANS-GFP-GUS gene fusion construct the PCR fragment obtained from PANS1FL-F and PANS1gus-R primer was cloned into pENTR/D-TOPO vector followed by LR reaction with pBGWFS7 destination vector [40].

The complementation construct was prepared by amplifying genomic DNA of *PANS*1 from Col-0 including 1006 bp upstream of ATG to 310 bp downstream of stop codon using PANS1FL-F and PANS1FL-R primers. The PCR fragment was cloned into pENTR/D-TOPO and mobilized into the pBGWFS7 destination vector.

### Microscopy

Immunostaining was performed as described in [38] using FLAG mouse monoclonal antibody (Sigma cat # F3165) at a 1:100 dilution and tubulin monoclonal antibody (sigma cat # T5168). Secondary antibodies were used at dilution of 1:100. Slides were mounted in 1 ug/ml DAPI in Vectashield (Vector Labs). Cells were imaged using a Zeiss Axio Imager.Z2 microscope equipped with dual camera (AxioCam MRm monochromatic, and AxioCam MRc colour) using a Plan-Apochromat 63× oil-immersion objective. False colouring was given through Axiovision software.

Meiotic chromosome spreads were carried out as described previously [41], with minor modifications [42]. Chromosomes were stained with DAPI (1 μg ml^−1^) and observed on a Zeiss Axio Imager.Z2 microscope (365 nm excitation; 420 nm long-pass emission), and FISH analysis was carried out according methods described earlier [43] with minor modifications [38]. The 180-bp centromeric pAL1 repeat was used to detect centromere sequences [22]. For probe preparation, a plasmid harboring two copies of the pAL1 repeat was subjected to PCR in the presence of Cy3-dATP (GE Healthcare), using PAL forward and reverse primers (Additional file 1: Table S2). Slides were observed under a Zeiss Axio imager microscope under 63× oil immersion objective, using an 550 nm excitation and 570 nm long-pass emission filter for Cy3. For pollen viability was examined using Alexander staining [44] and observed at 10x in DIC mode using a Zeiss Axio Imager.Z2 microscope. Images were captured using an AxioCam MRc camera.

Tissues from PANS1-GFP-GUS fusion transgenic plants were analysed by GUS staining as described previously [37].

Editing and annotation was done using Photoshop 6.0 (Adobe, http://www.adobe.com).

### Availability of supporting data

The data supporting the findings of this article are included within the article and in the additional files.

## Additional file

Additional file 1:
**Figure S1.** The *pans1* mutant produces microspores containing micronuclei. **Figure S2.** Centromeric FISH signal at zygotene. **Figure S3.** PANS1-FLAG immunolocalization detects expression in meiosis prophase I. **Figure S4.** *tam1-2* suppresses *pans1* sterility. **Table S1.** Seed set and pollen viability in *tam1, pans1,* and *pans1 tam1* double mutant. **Table S2.** List of primers used in study.

## Abbreviations

PANS1: PATRONUS1
APC/C: Anaphase promoting complex/cyclosome
osd1: Omission of second division 1
Scc1: Sister chromatid cohesion 1
SCC3: Sister chromatid cohesion 3
Rad21: Radiation sensitive 21
Rec8: Recombination defective 8
PP2A: Protein phosphatase 2A
SGO: SHUGOSHIN
WAPL: Wings apart like
GFP-GUS: Green fluorescent protein-beta glucuronidase
tam1: tardy asynchronous meiosis 1
CMR1: COPPER MODIFIED RESISTANCE 1
CDC27b: CELL DIVISION CYCLE 27b
CDC20.1: CELL DIVISION CYCLE 20.1
MIS12: MINICHROMOSOME SEGREGATION 12
