# Supplementary material for: PATRONUS1 is expressed in meiotic prophase I to regulate centromeric cohesion in Arabidopsis and shows synthetic lethality with OSD1
Source: BMC Plant Biol. 2015 Aug 14;15:201. doi: 10.1186/s12870-015-0558-6 (PMC4536785; doi:10.1186/s12870-015-0558-6)
Supplement: Additional file 1: — Figure S1. The pans1 mutant produces microspores containing micronuclei. Figure S2. Centromeric FISH signal at zygotene. Figure S3. PANS1-FLAG immunolocalization detects expression in meiosis prophase I. Figure S4. tam1-2 suppresses pans1 sterility. Table S1. Seed set and pollen viability in tam1, pans1, and pans1 tam1 double mutant. Table S2. List of primers used in study. [file 12870_2015_558_MOESM1_ESM.docx]

**Figure S1: The *pans1* mutant produces microspores containing micronuclei.** **(A)** Wild type microspore (**B**) *pans1-1*microspore (**C**) quantification of microspores in wild type and *pans1*. Scale bar represent 10 μm

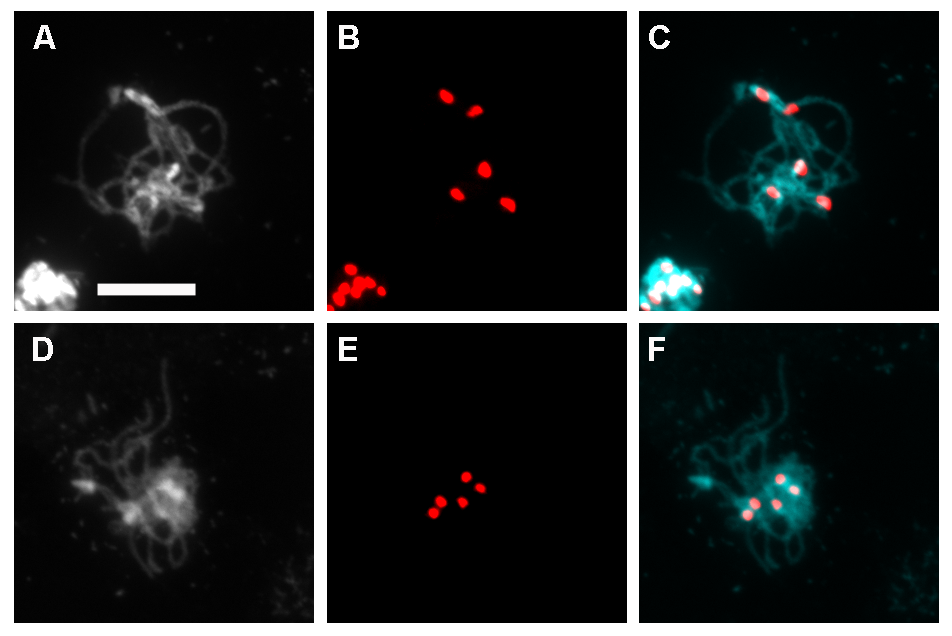


**Figure S2: Centromeric FISH signal at zygotene.** FISH of male meiotic chromosome spreads hybridized with a centromeric probe (red) at middle: left column showing DAPI (cyan); and right column: merged images of DAPI and the probe;. (**A-C**) *wild type* zygotene. **(D-F)** pans1 zygotene. Scale bar represent 10 μm

**
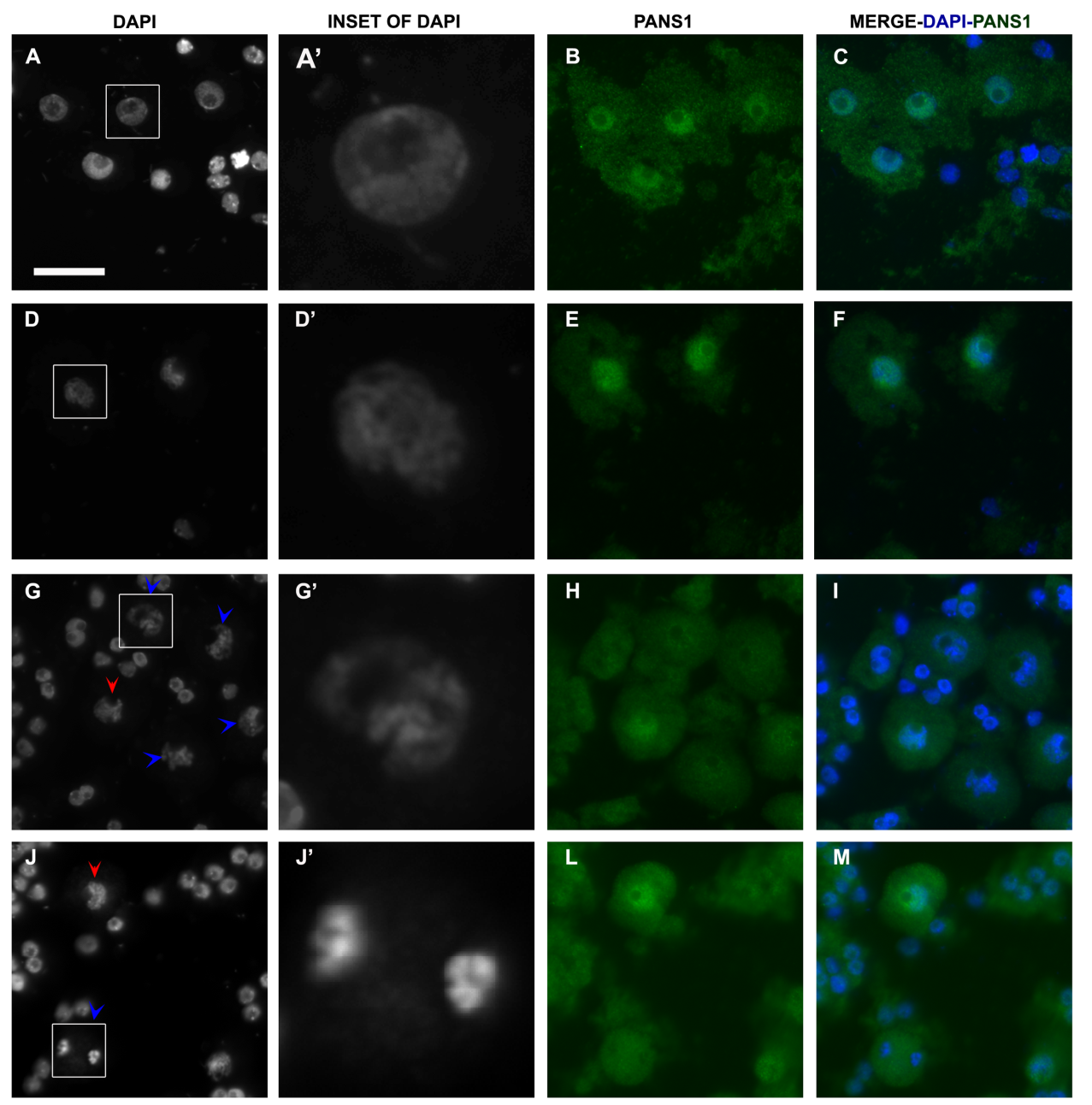
**

**Figure S3:** **PANS1-FLAG immunolocalization detects expression in meiosis prophase I.** Meiocytes at different stages within the same field show different levels of expression. Columns 1 and 2: DAPI. Column 3: PANS1-FLAG. Column 4: merged image. (**A-C**) Early prophase I. (**D-F**) Mid prophase I. **(G-I**) Mid prophase I (red arrowhead) plus late prophase I (blue arrowheads). **(J-L**) Mid prophase I (red arrowhead) plus Interkinesis (blue arrowhead) A’, D’, G’, and J’ show magnified images from the corresponding square box, as inset of DAPI. Scale bar represent 20 μm


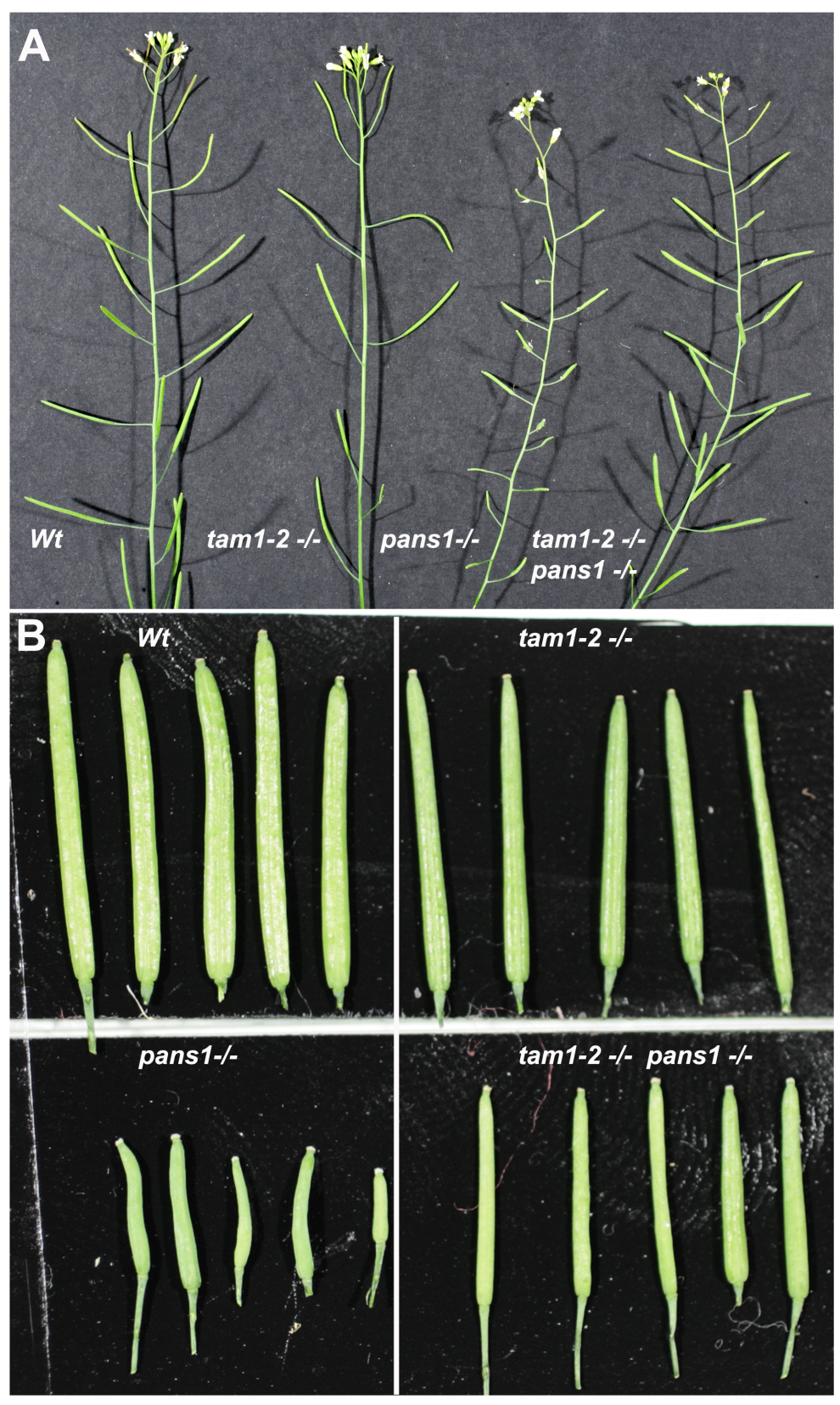


**Figure S4:**  ***tam1-2* suppresses *pans1* sterility**. **(A)**Wild type (Wt), *tam1-2* (-/-), *pans1-1*(-/-), and *tam1-2*(-/-) *pans1-1*(-/-) double mutant plants **(B)** Sliliques of Wild type (Wt), *tam1-2* (-/-), *pans1-1*(-/-), and *tam1-2*(-/-) *pans1-1*(-/-) double mutant.


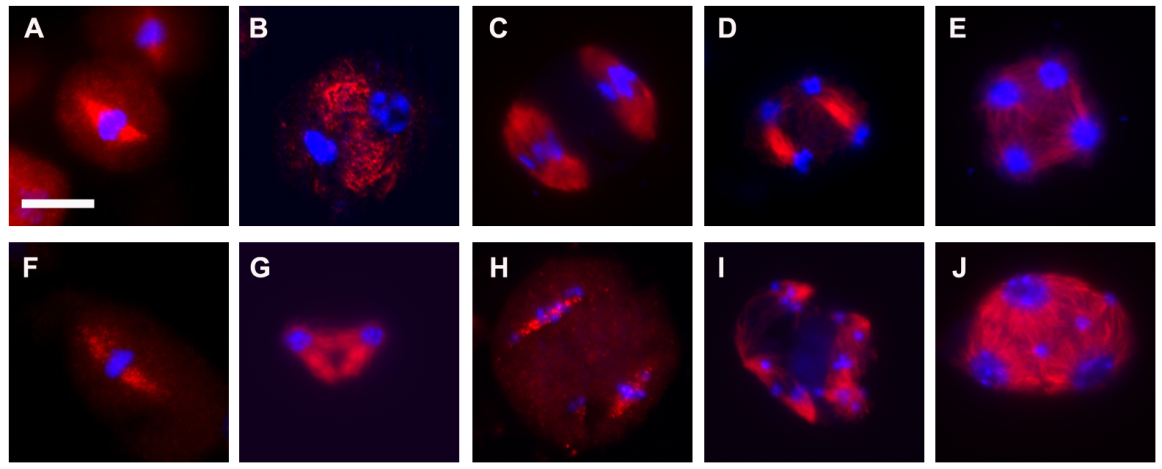


**Figure S5: Spindle abnormalities in *pans1.*** **(A-E)** wild type **(F-J)** *pans1*, (**A,F**) Metaphase I , (**B**) Telophase I of wild type compared with **(G)** Bent spindle phenotype seen in 11/58 meiocytes at Telophase I of *pans I.* **(C)** Metaphase II of wild type compared with **(H)** defective metaphase II anaphase II like spindle, (**D**) Anahase II of wild type compared with (**I**) multiple spindle phenotype of *pans I*. (**E**) Tetrad of wild type compared with **(J)** polyads of *pans I.* Scale bar represents 10 μm.

**Table S1: Seed set and pollen viability in *tam1, pans1,* and *pans1 tam1* double mutant.**

| Genotype | Average no. of missing seeds/silique | Average no. of aborted seeds/silique | Average no. of Viable  seeds/silique | Average no. of viable pollens /anther | Average no. of Dead pollens /anther |
| --- | --- | --- | --- | --- | --- |
| *Wild type* | 0.3 ± 0.5  (0.5%) | 0.4 ± 0.5  (0.7%) | 57.4 ± 2.4  (98.8%) | 489 ± 33.5  (100%) | 0.0 ± 0.0  (0.0%) |
| *tam1-2* | 0.8 ± 0.8  (1.5%) | 16.1 ± 2.1  (32%) | 33.5 ± 2.3  (66.5%) | 227 ± 18.7  (100%) | 0.0 ± 0.0  (0.0%) |
| *pans1-1* | 44.9 ± 4.3  (96.2%) | 0.5 ± 0.6  (1%) | 1.3 ± 1.4  (2.7%) | 41.9 ± 10.9  (20%) | 164 ± 44.8  (80%) |
| *pans1-1*  *tam1-2* | \| 12.1 ± 1.2 \| \| --- \| \| (27%) \| | \| 2.7 ± 1.0 \| \| --- \| \| (6%) \| | \| 30 ± 3.1  (66.9%) \| \| --- \| \|  \| | 155 ± 22.5  (84.8%) | 27.8 ± 11.3  (15.2%) |

**Table S2: List of primers used in study.**

| s.no. | primer name | Sequence 5’-3’ |
| --- | --- | --- |
| 1 | SALK_035661-LP | TGTTCTTCTTGCGATTGTTTG |
| 2 | SALK_035661-RP | CATGGAGTCGAGTTCTTCAGC |
| 3 | PANS1FL-F | CACCGTTGCTTGAACGGTCAGGAG |
| 4 | PANS1FL-R | CGGAACAGTCGCGAGGATAG |
| 5 | PANS1FL-NF | CACCGGACGTTAATTAGATATGG |
| 6 | PANS1cflag-R | AAGCTTGTCGTCATCGTCTTTGTAGTCGAAGAGGTCGTCAGAGTC |
| 7 | PANS1gus-R | CGAAGAGGTCGTCAGAGTC |
| 8 | F_PANS1qRT | CACTACTCTGCTGATCTC |
| 9 | R_PANS1qRT | GGAAGGCATCATAGAGCC |
| 10 | Pal_R | AGTCTTTGGCTTTGTGTCTT |
| 11 | Pal_F | TGGACTTTGGCTACACCATG |
| 12 | SALK_LB1.3 | ATTTTGCCGATTTCGGAAC |
| 13 | R_GAPCqRT | CAGTCTTCTGAGTAGCAGTGATTGA |
| 14 | F_GAPCqRT | AGCACGAATACAAGTCCGACCT |
| 15 | Osd1-3U | CACCTCTACGCGACATAACTC |
| 16 | 0sd1-3L | AGAAACCACCGAACTTGTGAAGA |
| 17 | Osd-1-3T-DNA | CTGGGAATGGCGAAATCAAGGCATC |
| 18 | Sail _505LP | TCAATCTGACCCGTCAATTTC |
| 19 | SAIL_505RP | TCTAGATCAGCAGGGAACCAC |
